# Bioaccumulation Patterns of Perfluoroalkyl Acids in an Estuary of the Ariake Sea, Japan

**DOI:** 10.1007/s00128-018-2282-z

**Published:** 2018-02-07

**Authors:** Jun Kobayashi, Yoshitaka Maeda, Yuki Imuta, Fumitaka Ishihara, Naoya Nakashima, Tomohiro Komorita, Takeo Sakurai

**Affiliations:** 1 Faculty of Environmental and Symbiotic Sciences, Prefectural University of Kumamoto, 3-1-100, Tsukide, Kumamoto, Kumamoto 862-8502 Japan; 2 Graduate School of Environmental and Symbiotic Sciences, Prefectural University of Kumamoto, 3-1-100 Tsukide, Kumamoto, Kumamoto 862-8502 Japan; 3 National Institute for Environmental Studies, 16-2 Onogawa, Tsukuba, Ibaraki 305-8506 Japan

**Keywords:** Bioaccumulation factor, Estuary, Perfluoroalkyl acids, Trophic level

## Abstract

To evaluate the bioaccumulation potential of perfluoroalkyl acids (PFAAs) in an aquatic food web, we measured the concentrations of nine PFAAs in the water and aquatic organisms from an estuary of the Omuta River, Japan. Average log bioaccumulation factors for all PFAAs ranged from 2.0 to 2.8. There was no positive correlation observed between PFAA carbon chain length and there was no evidence of trophic magnification demonstrated among the sample types collected. These results differed from the findings of previous studies in enclosed bodies of water, perhaps because river mouth–estuarine ecotones are more variable spatially and temporally and include some fish that are highly migratory. Further investigations of bioaccumulation factors will be needed to elucidate the tendency of amphiphilic chemicals to bioaccumulate in these river mouth–estuarine ecotones.

Perfluoroalkyl acids (PFAAs) such as perfluorooctanesulfonate (PFOS) have been found in water and organisms around the world due to their strong persistence and tendency to undergo bioconcentration. Since the restriction on the production and use of PFOS and its salts by the Stockholm Convention in 2009, concentrations of PFOS and perfluorooctanoic acid (PFOA) in aquatic environments have gradually decreased (Sakurai et al. 2016). However, increases in the concentrations of other PFAAs such as perfluorononanoic acid (PFNA) have been reported (Zushi et al. 2011). The magnitude of such increase depended on the conditions under which the PFAAs were used in an area. Therefore, monitoring of PFAAs other than PFOS and PFOA in aquatic environments is necessary to obtain a better understanding of their environmental behavior and bioaccumulation potential. Species-specific bioaccumulation patterns of PFAAs have been reported (Hong et al. 2015; Nakata et al. 2006). The objective of the present study was to determine the concentrations of PFAAs in water and aquatic organisms and to analyze their bioaccumulation characteristics. Estuaries provide habitat for a wide variety of organisms and are among the most important aquatic ecotones with respect to conservation of biodiversity.

## Materials and Methods

**Fig. 1:**
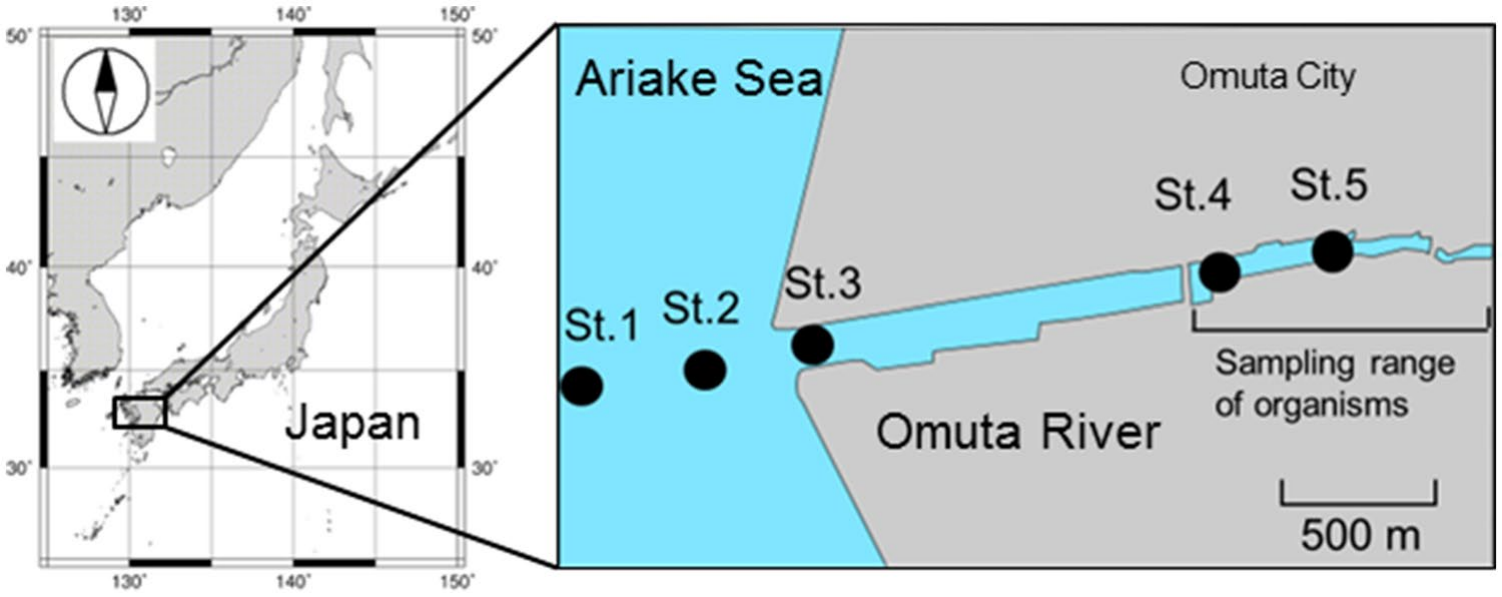
Sampling sites in the Omuta River mouth and estuary

Water samples were collected from five sampling stations along a transect of the Omuta River (Fig. 1) during the ebb tide in October 2012 and August 2013. At each station and each sampling, water samples from the surface layer (0–0.5 m depth) and the bottom layer (1 m above the bottom) were taken with a stainless steel bucket and a Van Dorn water sampler, respectively. A bottom-water sample at St. 5 in August 2013 was not collected because the water was too shallow; the total number of samples was therefore 19. The water samples were stored in polypropylene bottles that were pre-cleaned with methanol and cooled in ice during the cruise. After sampling, water samples were stored at a temperature below − 20°C until analysis.

Fish samples were collected with a cast net in the reach between St. 4 and upstream of St. 5, and snail samples were collected by hand around St. 4 in October 2012. Details of the sampling were described by Kobayashi et al. (2015). Species collected and analyzed for PFAAs included; sea snail (*Cerithidea rhizophorarum*, *n* = 10), javelin goby (*Acanthogobius hasta*, *n* = 3), yellowfin goby (*Acanthogobius flavimanus*, *n* = 1), grey mullet (*Mugil cephalus*, *n* = 3), and sea bass (*Lateolabrax* sp., *n* = 3).

Nine PFAAs (perfluoro-*n*-pentanoic acid, PFPeA; perfluoro-*n*-hexanoic acid, PFHxA; perfluoro-*n*-heptanoic acid, PFHpA; perfluoro-*n*-octanoic acid, PFOA; perfluoro-*n*-nonanoic acid, PFNA; perfluoro-*n*-decanoic acid, PFDA; perfluoro-*n*-undecanoic acid, PFUnDA; perfluorohexanesulfonic acid, PFHxS; and perfluorooctanesulfonic acid, PFOS) were analyzed. Water samples were filtered through a glass fiber filter (nominal pore size 0.45 µm) that had been pre-washed with methanol. The filtered water was used for the PFAA analyses. After spiking 500 mL of a water sample with 50 µL of a methanol solution containing 10 ng of ^13^C_4_-labeled internal standards (perfluoro-[1,2,3,4-^13^C_4_] octanesulfonic acid [MPFOS], Wellington Laboratories Inc., Guelph, Ontario, Canada), the water sample was passed through a solid phase extraction (SPE) cartridge Presep PFC-II (Wako Pure Chemical Industries, Ltd., Osaka, Japan) that had been pre-conditioned with 2 mL of methanol and then 2 mL of ultrapure water. The cartridge was eluted with 2 mL of methanol at a rate of about 10 mL/min. The eluates were collected in a 10-mL polypropylene tube. The eluate was concentrated under a gas stream of ultrapure N_2_ to a volume of 1 mL.

Whole fish were analyzed after removal of the stomach and gastrointestinal tract. The soft tissues of snails were analyzed as pooled samples. Two grams of homogenized sample were packed into stainless steel cells for extraction of the PFAAs with an accelerated solvent extractor (ASE200, Thermo Fisher Scientific Inc., Waltham, MA, USA) and spiked with 10 ng of ^13^C_4_-labeled internal standards (MPFOS). Samples were extracted using the ASE200 with a 10% methanol/water solution (v/v). Extracts were evaporated to approximately 2 mL in a rotary evaporator. The extract was diluted 100-fold with ultra-pure water for the clean-up procedure with SPE cartridges. The extract was passed through a Presep PFC-II cartridge connected with a Spelclean LC-Alumina-B SPE cartridge (Sigma-Aldrich, St. Louis, MO, USA) that was pre-eluted with 2 mL of methanol and then 2 mL of ultrapure water. The procedure after elution was the same as the procedure used for the water sample. PFAAs were identified and quantified using a high-performance liquid chromatograph with tandem mass spectrometry (Ultimate 3000/TSQ Quantum Access Max, Thermo Fisher Scientific). An aliquot of 10 µL of the sample was injected onto a Wakopak Wakosil-II 3C18RS column (2.0 mm i.d. × 150 mm length, 3 µm; Wako Pure Chemical Industries, Ltd.). Recovery rates of the nine PFAAs in recovery tests that involved standard additions to fish samples were as follows. PFHxS, 117% ± 33%; PFOS, 111% ± 18%; PFPeA, 68% ± 16%; PFHxA, 61% ± 7%; PFHpA, 118% ± 22%; PFOA, 117% ± 24%; PFNA, 92% ± 20%; PFDA, 87% ± 26%; and PFUnDA, 51% ± 18%. The trophic levels (TLs) of the organisms were based on the values reported by Kobayashi et al. (2015). The TLs were determined using nitrogen stable isotope ratios, and the snail was designated as a secondary consumer (TL = 2).

A bioaccumulation factor (BAF) was calculated based on Eq. 1. Here, BAF refers to ratio of PFAA concentration in aquatic organisms (*C*_B_ in ng/kg wet weight [ww], which is 10^3^ × *C*_B_ in ng/g wet weight) to the dissolved form of PFAAs in water (*C*_W_; ng/L). Average concentrations were used in the calculation.

1 $${\text{BAF }}={C_{\text{B}}}/{C_{\text{W}}},$$

The Student’s *t* test (*α* = 0.05) was used to test whether differences in PFAA concentrations between surface water and bottom water at each location were statistically significant. As for the correlations between PFAA concentrations and salinity in water and between PFAA concentrations and TL in organisms, a test for no correlation (*α* = 0.05) was used.

## Results and Discussion

Concentrations of PFAAs in the water are listed in Table 1. Average concentrations of perfluoroalkyl carboxylic acids (PFCAs) decreased as the length of the carbon chain increased. In the water samples, PFPeA (15 ng/L, *n* = 19) showed the highest concentration among the PFCAs, followed by PFHxA (11 ng/L), PFHpA (5.9 ng/L), PFOA (3.4 ng/L), and PFNA (2.6 ng/L). The concentrations of PFDA (< 0.59 ng/L) and PFUnDA (< 0.22 ng/L) were below the instrument detection limit. Of the perfluoroalkyl sulfonic acids (PFSAs), average concentrations of PFHxS and PFOS were 12 and 2.6 ng/L, respectively (*n* = 19). As for difference among sites, in October 2012 concentrations of PFPeA, PFOA, and PFNA decreased with increasing salinity from St. 5 to St.1. No statistically significant correlations were observed between concentration and salinity for the other compounds in October 2012 and for all compounds in August 2013 (*r* = 0.017–0.57; *p* = 0.085–0.97). The Student’s *t* test did not indicate any statistically significant difference in PFAA concentrations between surface water and bottom water at each location (*t* = − 0.98–1.06; *p* = 0.33–0.98). The concentrations of these PFCAs and PFSAs in this study were lower than the corresponding concentrations in water collected from rivers in the Tokyo Bay basin (Zushi et al. 2011), from rivers discharging directly into Tokyo Bay (Sakurai et al. 2016), and from coastal areas of Korea (Hong et al. 2015). Changes of the dominant compounds in the sampling areas and sampling year were observed in Tokyo Bay and coastal areas of Korea. In the case of river waters in the Tokyo Bay basin, PFOA was dominant in 2008 (Murakami et al. 2008), but by 2011 PFNA was dominant because PFNA had been produced selectively in industrial production of PFCA in Japan (Zushi et al. 2011). The concentration of PFHxS was the highest among the PFSAs and that of PFPeA was the highest among the PFCAs in the present study, although PFOA and PFNA have been reported to be dominant in previous studies (Murakami et al. 2008; Zushi et al. 2011). This result suggests that shorter chain compounds may be used instead of PFOS and PFOA in this area.

**Table 1:** Concentrations of PFAAs in water and organism samples

	Water^a^ (*n* = 19)	Sea bass (*n* = 3)	Grey mullet (*n* = 3)	Yellowfin goby (*n* = 1)	Javelin goby (*n* = 3)	Snail^b^ (*n* = 3)	Average of the five species
	ng/L	ng/g-ww	ng/g-ww	ng/g-ww	ng/g-ww	ng/g-ww	ng/g-ww
PFHxS	12 ± 16	7.3 ± 2.1	< 0.77	5.1	12 ± 7.7	5.2 ± 0.93	6.0 ± 4.5
PFOS	2.6 ± 1.5	1.0 ± 0.59	2.7 ± 2.2	1.5	2.6 ± 2.1	0.007 ± 0.011	1.5 ± 1.1
PFPeA	15 ± 15	1.5 ± 1.0	< 0.62	2.2	1.9 ± 2.1	1.2 ± 0.70	1.4 ± 0.9
PFHxA	11 ± 10	3.7 ± 1.8	< 0.25	6.8	2.8 ± 2.0	4.6 ± 0.61	3.6 ± 2.5
PFHpA	5.9 ± 4.4	0.70 ± 0.087	< 0.24	1.6	1.0 ± 0.46	0.31 ± 0.20	0.72 ± 0.62
PFOA	3.4 ± 2.9	1.0 ± 0.79	1.3 ± 0.62	8.3	0.53 ± 0.12	0.91 ± 0.44	2.4 ± 3.3
PFNA	2.6 ± 1.8	0.37 ± 0.36	0.88 ± 0.46	0.90	0.75 ± 0.50	1.1 ± 0.40	0.79 ± 0.26
PFDA	< 0.59	0.31 ± 0.18	0.62 ± 0.16	0.50	0.41 ± 0.025	< 0.29	0.37 ± 0.23
PFUnDA	< 0.22	< 0.11	0.67 ± 0.32	0.96	0.41 ± 0.17	< 0.11	0.41 ± 0.42
TL^c^	‒	3.5 ± 0.5	1.8 ± 0.7	2.6 ± 0.5	2.8 ± 0.6	2.0	‒

Values are average ± standard deviation

^a^Dissolved phase

^b^Composite sample

^c^Trophic levels (Kobayashi et al. 2015) calculated from nitrogen stable isotope ratios

Concentrations of PFAAs in each organism are listed in Table 1. Among the PFCAs, PFHxA (3.6 ng/g-ww) was present at the highest average concentration of the five species; it was followed by PFOA (2.4 ng/g-ww), PFPeA (1.4 ng/g-ww), PFNA (0.79 ng/g-ww), PFHpA (0.72 ng/g-ww), PFUnDA (0.41 ng/g-ww), and PFDA (0.37 ng/g-ww). The average concentrations of PFHxS and PFOS in the five species were 6.0 and 1.5 ng/g-ww, respectively. These concentrations were lower than those in fish collected in esturine and coastal areas of Korea (Hong et al. 2015) and in lake trout collected from the Great Lakes (Furdui et al. 2007). Differences in PFAA concentrations among organisms may be due to differences in habitat, concentrations of PFAAs in environmental media, and species-specific physiological factors such as respiration rates, feeding rates, metabolic rates, egestion rates, and growth rates (Falk et al. 2015). Although gobies and grey mullet are benthic fish, yellowfin gobies mainly consume polychaete and mysid, whereas grey mullets consume benthic microalgae in the study area.

**Fig. 2:**
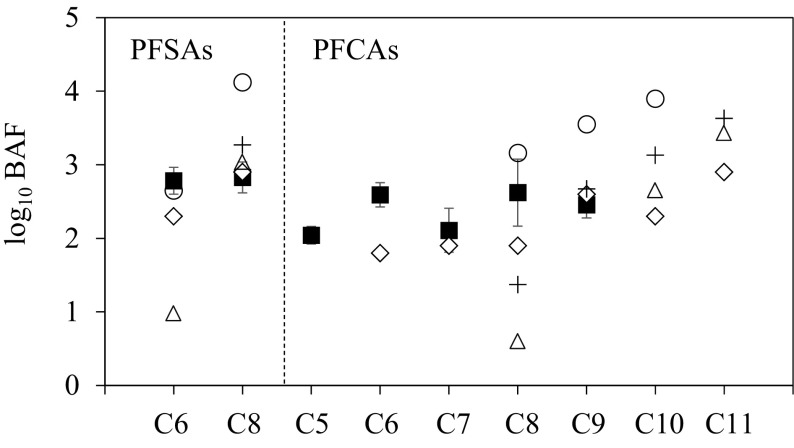
Comparison of average BAFs of PFAAs between this study and previous studies. Filled square, Omuta River estuary (the present study, averages and standard deviations of log BAF values for five species); opened circle, Great Lakes (Furdui et al. 2007); opened rhombus, coastal areas of Korea (Hong et al. 2015); plus, Taihu Lake (Xu et al. 2014); opened triangle, laboratory experimental bioconcentration factor in rainbow trout (Martin et al. 2003)

The BAF values were relatively similar among species and compounds. Average ± SD values of log BAF for each of the five species were as follows: PFHxS, 2.8 ± 0.2; PFOS, 2.8 ± 0.2; PFPeA, 2.0 ± 0.1; PFHxA, 2.6 ± 0.2; PFHpA, 2.1 ± 0.3; PFOA, 2.6 ± 0.5; and PFNA, 2.5 ± 0.2 (Fig. 2). The BAF values of PFDA and PFUnDA could not be calculated because the concentrations in the water were below the limits of detection for these compounds. The BAF values in this study were comparable to values determined for samples collected from estuarine and coastal areas of Korea (Hong et al. 2015) and Lake Taihu in China (Xu et al. 2014) but lower than those observed in the Great Lakes (Furdui et al. 2007). The fact that the BAFs in this study were lower than Great Lakes BAFs possibly reflects differences of the tissue analyzed. Muscle tissue was used for calculating BAFs in this study and the studies of Hong et al. (2015) and Xu et al. (2014), whereas whole body values were used in the study by Furdui et al. (2007). BAF for muscles would be lower than that for whole body because PFAA concentration in muscles is lower than that in other tissues, such as those of the liver (Martin et al. 2003). An increase of the BAF values with carbon chain length was not evident in this study over the limited range of carbon chain lengths (5–9). This result is consistent with those of Hong et al. (2015) in coastal areas of Korea. However, in a laboratory experiment (Martin et al. 2003) and in the field survey of Lake Taihu (Xu et al. 2014), increases of BAF values from short-carbon-chain compounds to long-carbon-chain compounds were observed (Fig. 2), in contrast to the results of this study. There was no correlation between the concentration of each PFAA and the trophic level of each organism in this study, although a positive correlation between these variables for biological samples collected from Lake Taihu (Fang et al. 2014). In an aquatic ecotone that includes an estuary and a river mouth, there can be wide spatial and temporal variance in PFAA concentrations, and study species such as the sea bass can migrate widely into and out of estuary habitats. Also, long-carbon-chain compounds such as PFDA and PFUnDA have higher uptake rate constants (Martin et al. 2003), and their concentrations require a longer time to reach steady state in fish. However, the number of sample and species were limited in this study. This limitation may affect the analysis of trophic magnification. Further investigations of PFAA bioaccumulation and trophic magnification factors in estuarine habitats will help to elucidate factors that contribute to the tendency of these amphiphilic compounds to bioaccumulate in aquatic species.
